# Genetic Manipulation of a Lipolytic Yeast *Candida aaseri* SH14 Using CRISPR-Cas9 System

**DOI:** 10.3390/microorganisms8040526

**Published:** 2020-04-07

**Authors:** Zool Hilmi Ibrahim, Jung-Hoon Bae, Sun-Hee Lee, Bong Hyun Sung, Ahmad Hazri Ab Rashid, Jung-Hoon Sohn

**Affiliations:** 1 Synthetic Biology & Bioengineering Research Center, Korea Research Institute of Bioscience and Biotechnology (KRIBB), 125 Gwahak-ro, Yuseong-gu, Daejeon 34141, Korea; abedozo@gmail.com (Z.H.I.); hoon@kribb.re.kr (J.-H.B.); werfg@kribb.re.kr (S.-H.L.); bhsung@kribb.re.kr (B.H.S.); 2 Department of Biosystems and Bioengineering, KRIBB School of Biotechnology, Korea University of Science and Technology (UST), 217 Gajeong-ro, Yuseong-gu, Daejeon 34113, Korea; 3 Industrial Biotechnology Research Centre, SIRIM Berhad, No.1, Persiaran Dato’ Menteri, Section 2, P.O. Box 7035, 40700 Shah Alam, Malaysia; ahazri@sirim.my

**Keywords:** *Candida aaseri*, CRISPR, genetic manipulation, acyl-CoA oxidase

## Abstract

A lipolytic yeast *Candida aaseri* SH14 that can utilise long-chain fatty acids as the sole carbon source was isolated from oil palm compost. To develop this strain as a platform yeast for the production of bio-based chemicals from renewable plant oils, a genetic manipulation system using CRISPR-Cas9 was developed. Episomal vectors for expression of Cas9 and sgRNA were constructed using an autonomously replicating sequence isolated from *C. aaseri* SH14. This system guaranteed temporal expression of Cas9 for genetic manipulation and rapid curing of the vector from transformed strains. A β-oxidation mutant was directly constructed by simultaneous disruption of six copies of acyl-CoA oxidases genes (*AOX2*, *AOX4* and *AOX5*) in diploid cells using a single sgRNA with 70% efficiency and the Cas9 vector was efficiently removed. Blocking of β-oxidation in the triple AOX mutant was confirmed by the accumulation of dodecanedioic acid from dodecane. Targeted integration of the expression cassette for *C. aaseri* lipase2 was demonstrated with 60% efficiency using this CRISPR-Cas9 system. This genome engineering tool could accelerate industrial application of *C. aaseri* SH14 for production of bio-based chemicals from renewable oils.

## 1. Introduction

Yeasts are unicellular eukaryotes with a long history of use in the biopharmaceutics and food industries [1,2]. Owing to their robust physiology in harsh conditions, such as elevated temperatures and low pH, many different yeasts have also been explored for the production of renewable bio-based chemicals to replace environmentally detrimental petrochemicals [3]. Bio-based production of long-chain dicarboxylic acids for polymer-based industries, including dodecanedioic acid (DDDA, C12), sebacic acid (C10), suberic acid (C8) and adipic acid (C6), has attracted considerable interest as a sustainable alternative to petroleum-based chemicals. Among them, adipic acid (1,6 hexanedioic acid) is an important building block for the manufacture of nylon 6,6, thermoplastic polyurethane resins, plasticisers, adhesives and synthetic lubricants [4]. The estimated global production of adipic acid in 2016 was 3.3 million tons, with an annual growth of 3% to 3.5% per year [5,6].

For bio-based production of adipic acid from renewable feedstocks, several methods have been developed in microorganisms. *Escherichia coli* and *Saccharomyces cerevisiae* were engineered to produce adipic acid from glucose by introduction of the reverse adipate-degradation pathway [7] and cis, cis-muconic acid pathway [8], respectively. Alkanes or fatty acids can be converted to the corresponding dicarboxylic acids by terminal oxidation of the ω-oxidation pathway and then used as carbon source via β-oxidation pathway in yeast. Different dicarboxylic acids were produced from alkanes or fatty acids by blocking or engineering of the β-oxidation pathway of a pathogenic yeast *Candida tropicalis* and the conversion efficiency was much improved by amplification of the ω-oxidation pathway [9]. Therefore, to produce long-chain dicarboxylic acids from renewable plant oils using microbial cell factories, a safe and robust strain that is able to utilise various plant oils as carbon sources and tolerant to organic acids is desirable [10,11].

A lipolytic yeast, *Candida aaseri* SH14, was previously isolated from the compost of oil palm empty fruit bunches for the production of bio-based chemicals [12]. The type strains of *Candida aaseri* (syn. *Candida butyri*) were isolated from butter by Nakase [13]. Until now, there have been few reports about this strain. A few years ago, *C. pseudoaaseri* closely related to *C. aaseri* was isolated from a clinical sample of an immunocompromised cancer patient on the basis of the nucleotide divergence [14]. Unlike *C. aaseri,* which is susceptible to all tested antifungals, *C. pseudoaaseri* was resistant to flucytosine and distinguished from *C. aaseri* by its colony morphology. Since *C. aaseri* SH14 strain shows outstanding lipolytic activity which derives from lipases (Figure S1), this strain can utilize various fatty acids from plant oils directly and long-chain alkanes as sole carbon sources and is resistant to high concentrations of organic acids (Figure S2). Thus, *C. aaseri* SH14 could be a microbial factory for the production of dicarboxylic acids, including adipic acid and other renewable chemicals, from plant oils.

However, this potential is limited by the lack of knowledge of the molecular mechanisms of *C. aaseri* SH14. Recently, we identified eight putative genes for lipase from this diploid strain in the draft genome sequencing and found that this strain belongs to the CTG:Ser group of *Candida* species [12]. Genetic engineering tools are further required to develop this strain as a platform host for the production of bio-based chemicals. Traditionally, the URA blast and Cre/LoxP systems have been used for multiple gene disruption in yeast [15,16]. However, these systems are suitable only for the metabolic engineering of established haploid strains having high homologous recombination activity, such as *S. cerevisiae, Schizosaccharomyces pombe* and *Kluyveromyces lactis*. Diploid or polyploid industrial yeasts are rarely suitable because the simultaneous modification of multiple alleles is an extremely inefficient and time-consuming way to obtain a null mutant. The discovery of clustered regulatory interspaced short palindromic repeats (CRISPR) system in bacteria [17] has been followed by its widespread use for numerous applications related to functional genomic studies in various organisms owing to its high efficiency, ease of operation and more rapid operation [18,19,20]. Several groups of organisms have been genetically edited using the modified type II CRISPR system [21,22,23]. The system is composed of a CRISPR-associated nuclease (Cas9) and a synthetic guide RNA (sgRNA) that confers target sequence specificity to Cas9 [24].

The CRISPR-Cas9 system has been applied in many different yeasts [25,26]. The core challenge of CRISPR-Cas9 for application in nonconventional yeast is how to express Cas9 and sgRNA. Generally, the expression vector for Cas9 and sgRNA is preferentially integrated into the genome if natural plasmid is not available. The first application of CRISPR-Cas9 in nonconventional yeast was in *C. albicans* [27]. A modified Cas9 and sgRNA expression cassette was integrated into a specific locus of the genome using a recyclable nourseothricin N-acetyl transferase (*NAT1*) cassette. After modification of the target genes, flippase was further expressed to remove the genome-integrated *NAT1* and Cas9 genes. In this version, the removal of genomic Cas9 was not efficient enough. A plasmid based CRISPR-Cas9 system was successfully developed in the nonconventional yeast *C. parapsilosis* [28]. Transient expression of Cas9 using a plasmid with an autonomously replicating sequence (ARS) obtained from the genome of *C. parapsilosis* has enabled the efficient multiple editing of target genes. Unlike these strains, Cas9 expression vectors of *S. cerevisiae* were directly used for genome engineering of *C. glabrata* without modifications [29]. Our initial approach using a plasmid system of *S. cerevisiae* in *C. aaseri* SH14 was not successful. There might be many unknown factors determining the efficiency of gene editing using CRISPR-Cas9 in nontraditional *Candida* yeasts due to the broad spectrum of *Candida* spp. Consequently, it is necessary to develop species-specific CRISPR-Cas9 systems for efficient genome engineering of *C. aaseri* SH14.

Here, we have developed a CRISPR-Cas9 system for genetic manipulation of *C. aaseri* SH14. The system consists of *Candida*-compatible Cas9 gene, *ARS*, gRNA expression cassette and nourseothricin (NTC) resistance selection marker in a single episomal plasmid. Multiple gene disruption, targeted integration of expression cassette and rapid curing of CRISPR-Cas9 plasmid were successfully demonstrated.

## 2. Materials and Methods

### 2.1. Strain, Chemicals and Media

*C. aaseri* SH14 and their mutant strains were cultured in YPD (1% yeast extract, 2% Bacto peptone and 2% glucose). Selection of URA+ transformants was performed on a synthetic complete medium lacking uracil (SC-ura; 0.67% yeast nitrogen base without amino acids, 0.077% ura dropout supplement, 2% glucose and 2% Bacto agar). For antibiotic media, YPD was supplemented with an appropriate concentration of nourseothricin (NTC, Sigma-Aldrich, St. Louis, MO, USA) or hygromycin (Sigma-Aldrich). SC+5-FOA contained SC supplemented with 0.01% (*w*/*v*) uracil (Sigma-Aldrich) and 0.05% 5-FOA (Duchefa Biochemies, Haarlem, The Netherlands). For SC with methyl laurate (SC+ML), glucose was replaced with 1% (*v*/*v*) methyl laurate (TCI Chemicals, Tokyo, Japan) equilibrated at pH 7.0 with NaOH. Lipase secreting transformants were screened using YP supplemented with 1% tributyrin (Sigma-Aldrich). *Escherichia coli* DH5α [F- lacZΔM15 hsdR17(r-m-) gyrA36] was used for general recombinant DNA techniques. All other chemicals were purchased from Sigma-Aldrich. Restriction enzymes were purchased from New England Biolabs (Ipswich, MA, USA). DNA purification was conducted using the Wizard® SV Gel and PCR Clean-Up Systems (Promega, Madison, WI, USA).

### 2.2. Cloning of ARS Element and Construction of Episomal CRISPR-Cas9 Vector

To obtain genomic ARS elements of *C. aaseri* SH14, a *C. aaseri* SH14 genomic library was constructed. The genomic DNA was partially digested with *Sau*3AI and DNA fragments ranging from 1.5 kb to 3 kb were cloned into the *Bam*HI site of pBluescriptII KS (+) containing the *NAT1* under the control of the *URA3* promoter and terminator. Plasmid digestion analysis showed that >80% clones contained average DNA fragments of 1.2 kb and approximately 3.7 × 10^8^ clones were screened for ARS elements. This library provided a 1.4 × coverage of the *C. aaseri* SH14 genome size. Plasmids were rescued from *C. aaseri* SH14 transformants that survived on the selective medium by back-transformation of *E. coli* with the total DNA harvested from *C. aaseri* SH14 transformants. For the construction of the CRIPSR-Cas9 vector (pAN-Cas9), we designed the CRISPR-Cas9 expression system together with sgRNA containing a target-specific sequence. For preparation of the Cas9 gene, the sequence retrieved from p414-TEF1p-Cas9-CYC1t [30] was edited based on the optimised codon of *C. aaseri* and synthesised with nuclear localization signal (NLS) by Bioneer (Daejeon, Korea). For the constitutive expression of *Cas9*, Cas9-NLS was flanked with the *GAPDH* promoter and terminator and cloned into the *XbaI and Xho*I sites of pBluescript-II KS (+) plasmid containing ARS. 

For the construction of the sgRNA expression cassette, a fragment fused with HH ribozyme, gRNA scaffold and HDV ribozyme was synthesised as previously described [31]. *TEF1* promoter and the *CYC1* terminator were used for transcription of the HH-sgRNA scaffold-HDV. The sgRNA target sequences (20bp) were incorporated into the pAN-Cas9 vector by overlap extension PCR (Figure S3). Sequences of constructed vector were confirmed by Genotech (Daejeon, Korea). All primers and vectors used in this study were listed in Tables S1 and S2.

### 2.3. Transformation and Characterization of Transformants

The transformation method described by Kondo et al. [31] was applied to *C. aaseri* SH14 with a slight modification. Cells were grown in YPD medium until the optical density at 600 nm (OD_600_) reached 1.0 and then were harvested by centrifugation at 3500 rpm for 20 min. Cells were suspended into transformation buffer containing 5 mM lithium acetate, 0.5 M sorbitol, 10 mM Tris-HCl (pH 7.4) and 1 mM EDTA, and then 0.01 vol of freshly prepared 1 M dithiothreitol (DTT) was added. After one-hour incubation at room temperature, cells were washed with 1 M sorbitol three times and resuspended in 0.5 mL of 1 M sorbitol for transformation. For each transformation, 0.5 µg of plasmid DNA was mixed with 100 µL of competent cells in a 2 mm electroporation cuvette and incubated on ice for 10 min. Electroporation was performed using the Gene-pulser II (Bio-Rad, Hercules, CA, USA) at 2.25 kV, 50 µF and 200 Ω. After electroporation, 1 mL of YPD containing 1M sorbitol was added and incubated at 30 °C overnight. Transformants carrying pAN-Cas9sgRNA plasmid were screened on YPD supplemented with 20 µg/mL NTC.

Positive transformants were confirmed by colony PCR. Randomly selected colonies were suspended in lysis buffer (10 mM sodium phosphate and 2 mg/mL lyticase) and incubated at 37 °C for 30 min. Then, proteinase K solution (2 mg/mL) was added to the mixture and incubated at 50 °C for 10 min followed by inactivation of the proteinase at 80 °C for 10 min. Cell lysate (1 μL) was used as a template for PCR analysis.

### 2.4. Screening of Recombinant C. aaseri SH14 Overexpressing CaLIP2

*C. aaseri* SH14 lipase2 (*CaLIP2*) expression cassette was designed and used in gene knock-in using Cas9 system. *CaLIP2* expression cassette contains *CaLIP2* gene fused with constitutive *TEF1* promoter of *C. aaseri* and *CYC1* terminator. For confirmation of additionally expressed *CaLIP2*, hexa histidine-tag was included at C-terminus of *CaLIP2*. This cassette was flanked with a 380 bp homology arms of *CaAOX2* gene near sgRNA targeting site at both ends. This fragment was used as a donor DNA. To integrate *CaLIP2* expression cassette into *CaAOX2* locus, pAN-Cas9-gAOX2 plasmid which targeted to a specific *AOX2* locus was co-transformed with donor DNA. Integration into the targeted locus was confirmed by PCR analysis using primers located on the cassette (LIP2R) and primers located at the upper site of *CaAOX2* locus (AOX2F).

Cells obtained after transformation of pAN-Cas9-gAOX2 plasmid and *CaLIP2* cassette were dotted onto YP supplemented with 1% tributyrin and incubated at 30°C to identify lipase secreting transformants based on the development of a clear halo. Lipase expressing transformants were cultured in YP broth for 40 h and culture broth was used to determine lipase activity, which was determined using *p*-nitrophenyl palmitate (*p*-NPP) as substrate [32]. The reaction mixture (50 mM Tris-HCl buffer, pH 8.0, 2 mM *p*-NPP and culture broth) was incubated at room temperature for 5 min and *p*-nitrophenol released by lipase was quantified using a spectrophotometer according to the absorbance at 405 nm. One unit of lipase activity was defined as the amount of enzyme needed to release 1 µmol of *p*-nitrophenol per minute. Transformants were cultivated in a 10 mL test tube containing 2 mL YPD broth medium for 40 h. Then, 0.6 mL of culture supernatant was precipitated with 0.4 mL of cold acetone and analysed by electrophoresis on 12% polyacrylamide gels under denaturing conditions by staining with Coomassie blue. The western blot analysis was performed using anti-His (Sigma Aldrich) after dilution of 1/1000 times. Protein samples were electrophoresed and transferred to nitrocellulose membrane using iBlot® 2 Dry Blotting System (Thermo Fisher Scientific) following manufacturer’s instructions. The reacting antibodies were detected with anti-mouse immunoglobulins conjugated to alkaline phosphatase (Sigma Aldrich).

### 2.5. Analysis of dodecanedioic Acid (DDDA)

Analysis of DDDA was performed using the method of Funk et al [33] with slight modifications. The culture broth was adjusted to pH 10 with sodium hydroxide and boiled for 5 min. Supernatant was acidified with 2N sulfuric acid and DDDA was extracted with 2 volume of MTBE (methly tert-butyl ether) for 2 h. Next step, extracted DDDA was mixed with 0.25 volume of trimethylsilyl trifluoroacetamide and silylated at 90°C for 50 min. DDDA and dodecane were measured by gas chromatography using HP5 column and Agilent 7890A gas chromatograph system (Agilent, Technologies, Yarnton, England) equipped with a flame ionization detector (FID) and a HP5 capillary column (30 m length, 0.32 mm ID, 0.25 µm film thickness). The He carrier gas flow was set to 3 mL/min and increased from 70 °C to 185 °C at 15 °C/min, then ramped to 300 °C at 30 °C/min and finally held at 300 °C for 2 min.

## 3. Results

### 3.1. Development of Transformation System for C. aaseri SH14

To develop a transformation system in *C. aaseri,* sensitivity to various antibiotics that are frequently used in transformation of yeast were analysed. Two antibiotics, NTC and hygromycin, which inhibited the growth of *C. aaseri* SH14, were selected and the minimum inhibitory concentrations were determined (Figure S4). Since *C. aaseri* SH14 showed higher sensitivity to NTC (10 μg/mL) than hygromycin (100 μg/mL), *NAT1* gene from *Streptomyces noursei* was employed as a selection marker gene. In addition, because the *NAT1* gene does not contain the CTG codon, which is predominantly translated as serine instead of leucine in CTG:Ser clade species, the gene was expressed under the control of *C. aaseri URA3* promoter without modification. To construct an episomal vector of *C. aaseri* SH14, ARS were isolated from a genomic library of *C. aaseri* SH14 constructed in the pBluescript II KS+ plasmid containing *NAT1* gene. *C. aaseri* SH14 was transformed with the genomic library without linearization and transformants were selected on YPD plates containing 20 μg/mL NTC. After screening of approximately 4 × 10^4^ transformants, two colonies were isolated and plasmids were retrieved from the transformants. Two different ARS elements were identified by DNA sequencing and designated ARS1 (1265 bp) and ARS2 (789 bp) (Figure S5).

**Figure 1:**
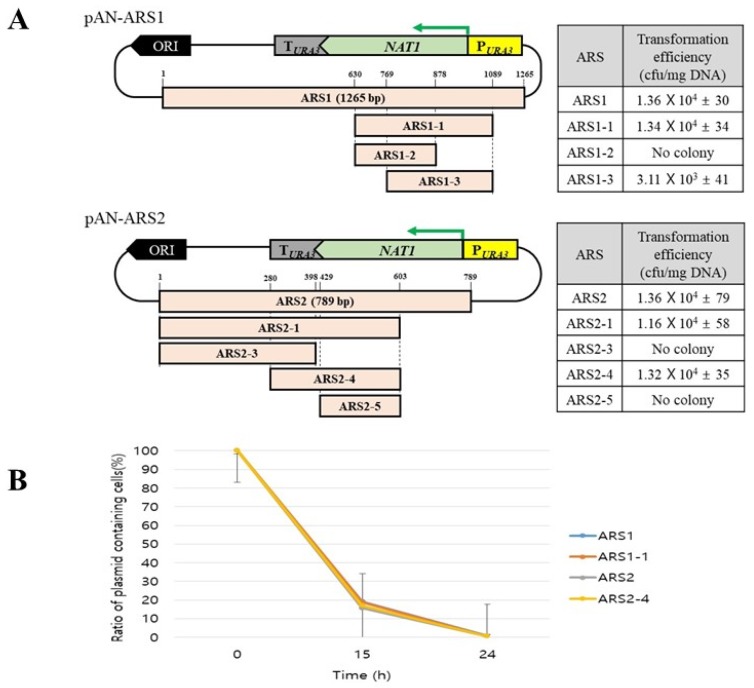
Determination of core domain of ARS1 and ARS2 and stability test of autonomously replicating sequence (ARS) plasmids. (**A**) Schematic diagram of ARS fragments and transformation efficiency. (**B**) Stability of different ARS plasmids. Mean values and standard deviations of triplicates are shown.

To define the core regions of ARS1 and ARS2 responsible for the replication of episomal plasmid, several plasmids containing each deleted ARS fragment were constructed, and their transformation efficiency was checked. Only ARS1-1 (460 bp) and ARS2-4 (324 bp) showed a comparable transformation efficiency with the full-length ARS elements (Figure 1A). The stability of each plasmid containing ARS1, ARS1-1, ARS2 and ARS2-4 was analysed in YPD medium without selection pressure. All tested plasmids completely disappeared after several generations under nonselective condition (Figure 1B). These results implied that the cloned ARSs are useful for the highly efficient transformation and simple elimination of plasmid containing Cas9 expression cassette in *C. aaseri* SH14. Considering the size of plasmid, the plasmid pAN-ARS2-4 containing ARS2-4 was selected for expression of Cas9.

### 3.2. Development of CRISPR-Cas9 Expression System for C. aaseri SH14

**Figure 2:**
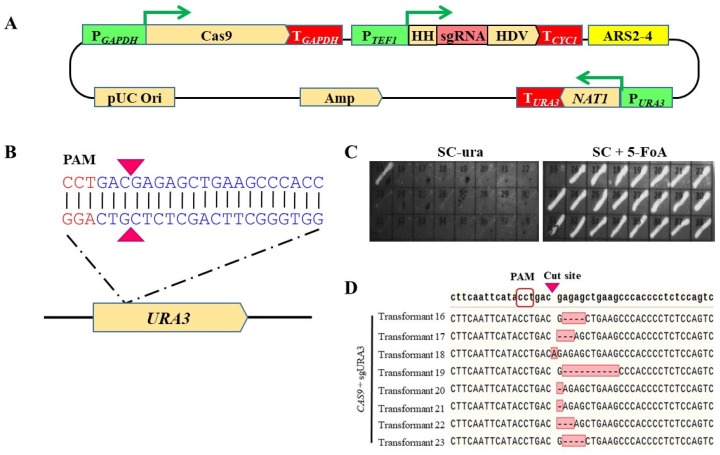
Targeted disruption of *CaURA3* using CRISPR-Cas9 system. (**A**) Schematic diagram of pAN-Cas9gRNA vector. HH; hammerhead ribozyme, HDV; and hepatitis delta virus ribozyme. (**B**) *CaURA3* target sequence generated by ATUM program. Arrowheads indicate Cas9 cleavage site. (**C**) Conformation of ura3 mutant phenotype on SC-ura and SC+5-FOA plates. (**D**) Sequence analysis of *CaURA3* from eight transformants. Insertions or deletions were indicated (red box).

For the development of an easily modifiable CRISPR-Cas9 system in *C. aaseri* SH14, a single vector system was designed for the expression of Cas9 and sgRNA. Considering the CTG:Ser clade of *C. aaseri*, a gene coding for a *Candida* codon optimised Cas9 (Figure S6, 84 CTG changed to CTT or TTA) fused with SV40 nuclear localization signal was synthesised and expressed under the control of the glyceraldehyde 3-phosphate dehydrogenase (*GAPDH*) promoter of *C. aaseri* SH14. For the expression of sgRNA, RNAP III promoters that do not modify the 5′ and 3′ end of RNA are typically required for correct formation of functional sgRNA. Since the confirmed RNAP III promoters are not available in *C. aaseri* SH14, the translational elongation factor1 (*TEF1*) promoter and two different self-cleaving ribozyme regions, hammerhead ribozyme (HH) and hepatitis delta virus (HDV), were used to make a mature sgRNA without post-transcriptional modifications [32]. The Cas9 and sgRNA cassettes were cloned into the pAN-ARS2-4 vector to construct a pAN-Cas9gRNA plasmid (Figure 2A).

As a proof of our system, the *CaURA3* gene was selected for targeted disruption. Synthesis of the *CaURA3* targeting sequence for sgRNA (gURA3) was designed using the ATUM CRISPR gRNA design tool (www.atum.bio/eCommerce/cas9/input) (Figure 2B). To eliminate off-target activity, the gRNA sequence generated from ATUM was confirmed by a similarity search against the *C. aaseri* genome database. *C. aaseri* SH14 was transformed using pAN-Cas9gURA3 without linearization and transformants were selected on YPD containing 20 μg/mL NTC. Twenty-four randomly selected transformants were transferred to both SC-ura and SC+5-fluoroorotic acid (FOA) plates. All except one of the transformants displayed the uracil auxotrophic phenotype growing on SC+5-FOA medium (Figure 2C). The sequences of *CaURA3* from eight transformants recovered from the SC+5-FOA plate were compared with the wild type sequence (Figure 2D). Seven transformants showed 1 to 10 base deletions and one contained one base insertion at the Cas9 cleavage site. Considering *C. aaseri* SH14 is diploid, two copies of *CaURA3* gene were disrupted with nearly 100% efficiency.

### 3.3. Deletion of Multiple Genes by a Single Guide RNA

**Figure 3:**
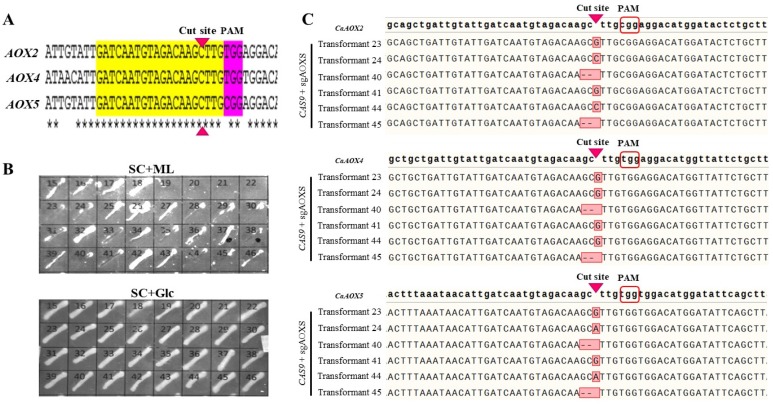
Multiple gene disruption by single gRNA. (**A**) Sequence alignment of *CaAOX2*, *CaAOX4* and *CaAOX5* genes. The conserved gRNA target sequence (20 bp) and PAM sequence were indicated. (**B**) Growth of mutants on methyl laurate (SC+ML) and glucose (SC+Glc) as a sole carbon source. (**C**) Sequence analysis of six different mutants. Insertions or deletions were indicated (red box).

In yeast, most of the fatty acid molecules are catabolized to the acetyl-CoA by β-oxidation pathway or are converted to dicarboxylic acids by ω-oxidation when β-oxidation is defective [33,34,35,36]. Therefore, to produce long-chain dicarboxylic acids from fatty acids, the β-oxidation pathway should be inactivated to block further oxidation to acetyl-CoA. β-oxidation is a cyclic degradation of a fatty acid catalysed by acyl-CoA oxidases (AOXs; EC: 1.3.3.6). Three AOX homologues, *CaAOX2*, *CaAOX4* and *CaAOX5*, were identified from the *C. aaseri* genome by BLAST analysis using known AOX genes. To find conserved regions that could be used as a common target for sgRNA, the nucleotide sequences of the three genes were aligned and a region having exactly the same 20 bp with different protospacer adjacent motif (PAM) sequences was selected (Figure 3A). The sgRNA target sequence of pAN-Cas9gRNA was replaced with this sequence to construct pAN-Cas9gAOX. *C. aaseri* SH14 was transformed using the plasmid and transformants were selected on YPD containing 20 μg/mL NTC. Since the mutants defective in β-oxidation were unable to utilize fatty acid as a sole carbon source, transformants were selected by replica plate analysis using SC+Glc and SC+ML containing 1% glucose and methyl laurate, respectively (Figure 3B). Twenty two of 32 transformants (70%) were unable to grow on SC+ML. To identify the sequence of AOX genes, specific primers located on each gene were used to amplify a region containing the sgRNA target site. Six randomly selected transformants per AOX gene showing a mutagenic phenotype were analysed. All tested AOX genes revealed indels at specific sgRNA targets (Figure 3C), suggesting that complete disruption of multiple genes of the six copies could be done with an efficiency of approximately 70% by a single transformation.

**Figure 4:**
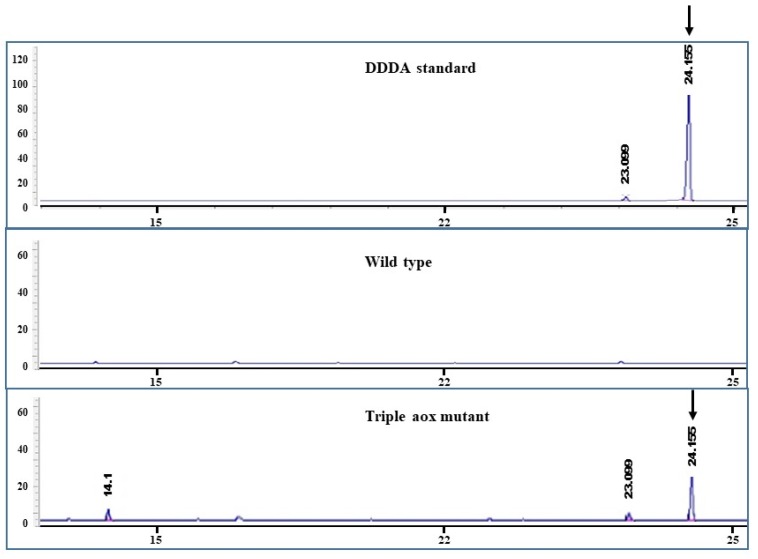
Gas Chromatography analysis for the detection of dodecanedioic acid (DDDA) from dodecane. DDDA peak was detected at 24.15 min from culture broth of triple *aox* mutant.

To confirm the induction of the ω-oxidation pathway in a triple *aox* mutant, conversion of dodecane to dodecanedioic acid (DDDA) was conducted using wild type and triple *aox* mutant (Figure 4). Contrary to the wild type, DDDA was found in the culture broth of the triple *aox* mutant, indicating complete inactivation of β-oxidation. Therefore, six copies of AOX genes in diploid were disrupted with 70% efficiency using a single sgRNA.

### 3.4. Cas9 Mediated Targeted Integration and Curing of pAN-Cas9gRNA Vector

**Figure 5:**
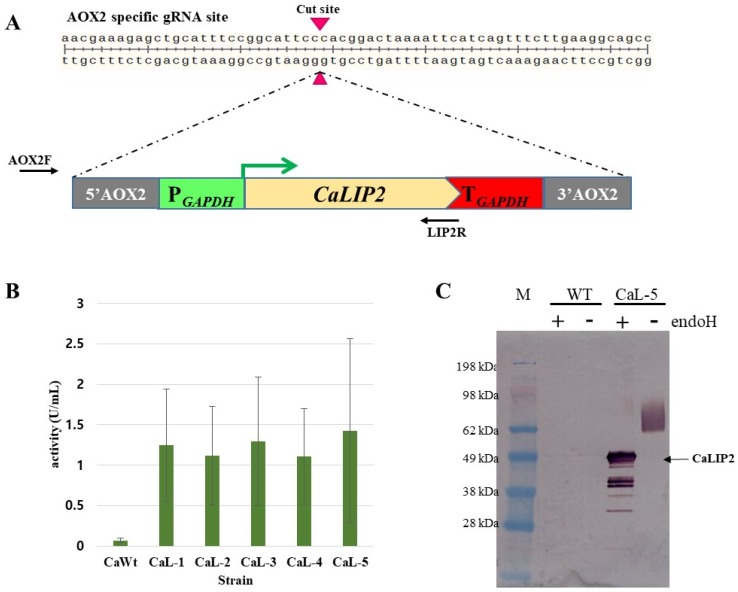
Targeted integration into specific locus by CRISPR-Cas9. (**A**) Schematic diagram of sgRNA target site and insertion of *CaLIP2* cassette via homologous recombination. (**B**) Lipase activity of selected transformants. (**C**) Confirmation of *CaLIP2* by western blot analysis.

Disruption of target genes using non-homologous end joining (NHEJ) and integration of recombinant genes into a specific locus by homologous recombination is an important tool for genetic engineering of industrial yeast. To confirm gene knock-in using the Cas9 system in *C. aaseri* SH14, the *C. aaseri* lipase2 (*CaLIP2*) expression cassette was integrated into the *CaAOX2* locus. The *C. aaseri* β-oxidation mutant was cotransformed with the Cas9 vector containing sgRNA expression cassette targeted to *CaAOX2* (pAN-Cas9gAOX2) and *CaLIP2* expression cassette containing 380 bp homology arms at both ends (Figure 5A, Figure S7). Overexpression of *CaLIP2* was distinguished by comparison of the halo zone generated by the wild type and transformants on tributyrin containing medium. Integration of the *CaLIP2* expression cassette into the *CaAOX2* locus was confirmed by PCR using primers located on the cassette (LIP2R) and primers located at the upper site of *CaAOX2* locus (AOX2F). Among the ten transformants showing expanded halo zones, six (60%) involved cassette integration via homologous recombination. Lipase activity of the positive transformants was over 10-fold higher than that of wild type (Figure 5B). After cultivation of a transformant showing the highest lipase activity, the culture broth was examined by western blot analysis (Figure 5C). Since CaLIP2 contains three putative glycosylation sites, a smear of protein bands larger than 62 kDa was detected in transformant CaL-5. After treatment with deglycosydase (Endo-H), mature CaLIP2 was detected as a 51.3 kDa band. Several protein bands smaller than mature CaLIP2 after deglycosylation might have resulted from proteolysis during cultivation. 

**Figure 6:**
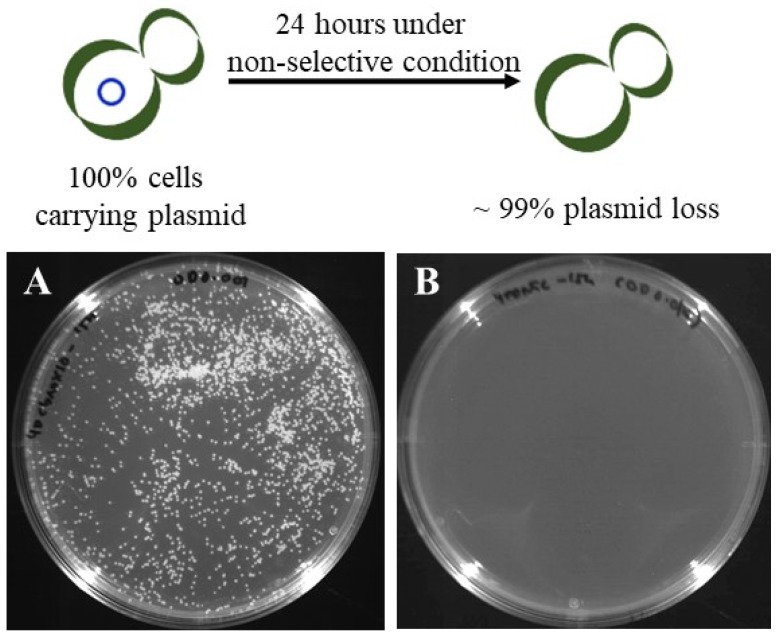
Complete loss of CRISPR-Cas9 plasmid from transformed cells under nonselective condition. Transformed cells harbouring pAN-Cas9gAOX2 vector was cultured in YPD broth without nourseothricin (NTC) for 24 h and an equal amount of cells was plated on YPD (**A**) and YPD+20 μg/mL NTC (**B**) plate.

For the repetitive use of the Cas9 to modify another target, easy removal of Cas9 and gRNA expression cassettes in the previously engineered cells is indispensable. To remove the pAN-Cas9gAOX2 plasmid from the recombinant strain, the strain was cultivated in YPD medium without NTC for 24 h and then an equal number of cells were plated on YPD and YPD+20 μg/mL NTC medium. As shown in Figure 6, no colonies developed on YPD+NTC medium, indicating the complete loss of the plasmid. Consequently, the Cas9 vector developed in this study was useful for the temporal expression of Cas9 nuclease sufficient for a genome editing and also for easy removal of Cas9 vector from the engineered cells.

## 4. Discussion

The success of the CRISPR-Cas9 system for genome editing of *S. cerevisiae* led to many studies of the use of the system for genetic manipulation of various industrial or non-conventional yeasts [37,38,39,40]. Especially, *Candida* spp has attracted considerable interest because it contains several strains showing a significant potential for biotechnological use [41] and clinically important pathogen [42]. Since the members of *Candida*, such as *C. albicans*, *C. glabrata* and *C. parapsilosis*, are clinically relevant pathogenic yeasts, the CRISPR-Cas9 system was developed to study virulence mechanisms [27,28,29]. Unlike these strains, *C. aaseri* SH14 is an environmental species isolated from the compost of oil palm empty fruit bunches. It utilises various fatty acids and alkanes as sole carbon sources. Therefore, this strain could potentially be a platform host for the microbial production of chemicals from renewable oils.

We developed a CRISPR-Cas9 system for the efficient genome engineering of *C. aaseri* SH14. The system consists of a *Candida*-compatible *Cas9* gene, sgRNA expression cassette, *NAT1* marker and ARS in an episomal vector. For easy elimination of the Cas9 and sgRNA cassettes after editing a target locus, both were incorporated in a single episomal vector rather than being integrated into the genome. Because there is no natural plasmid in *C. aaseri* SH14, we developed an artificial plasmid using ARS elements isolated from a genomic DNA library. Two ARS elements, ARS1 and ARS2, were isolated and their ARS core regions were determined. The core regions of ARS1 and ARS2 were enriched in A-T (>70%), which is a typical feature of ARS required for the initiation of replication. Artificial plasmids harbouring both ARSs greatly improved the transformation efficiency in *C. aaseri* SH14 but were stably maintained only under the selective condition.

Functional expressions of Cas9 and sgRNA are necessary for this system to work. Since *C. aaseri* SH14 is a CTG:Ser clade, all 84 CTG codons found in the *Cas9* gene were changed to CTT or TTA and were expressed under the control of a strong and constitutive *GAPDH* promoter. As found in other strains, expression of Cas9 protein was also detrimental to *C. aaseri* SH14 and severely affected growth (Figure S8). The negative effect of Cas9 on cell growth could be eliminated by the easy curing of the Cas9 plasmid from *C. aaseri* SH14. To express sgRNA without post-transcriptional modifications, RNA polymerase II promoter was used with a self-cleavage sequence, such as HH and HDV ribozymes.

As a proof of concept for multiple gene disruption using this system, disruption of three different AOX genes (six copies in diploids) was done using a single sgRNA. Since three AOX genes were found in *C. aaseri* SH14 for fatty acid assimilation through β-oxidation and they are highly conserved, a common target for sgRNA synthesis was selected to make the β-oxidation mutant by a single transformation of the sgRNA vector. All AOX genes of the selected transformants were abolished with indels. Selected strains showed a typical phenotype of β-oxidation mutants, namely the inability to grow on a fatty acid, such as methyl laurate or oleic acid, supplied as the sole carbon source. When dodecane was supplied as a carbon source, DDDA was detected only in the triple aox mutant implying inactivation of β-oxidation pathway. However, the conversion yield was very low compared to that of *C. tropicalis* [9]. In case of *C. tropicalis*, the ω-oxidation pathway was amplified by overexpression of the cytochrome P450 monooxygenase and NADPH-cytochrome reductase genes encoding the rate-limiting steps of the ω-oxidation pathway. Therefore, further engineering of the ω-oxidation pathway of *C. aaseri* SH14 triple aox mutant might be required to improve the conversion yield. From the genome sequence analysis, four genes with high similarity to cytochrome P450 monooxygenase were identified. Engineering of the ω-oxidation pathway *C. aaseri* SH14 using these candidates is underway.

Considering that *C. aaseri* SH14 is diploid, six copies of AOX genes were disrupted with 70% efficiency using a single sgRNA. Since β-oxidation is catalysed by several acyl-CoA oxidase isozymes, several AOX genes were identified in plants, animals and microorganisms. Especially, in the yeast *Yarrowia lipolytica*, five AOX genes have been identified [43]. Besides, designing of common sgRNA is possible because the nucleotide sequence of these genes is highly conserved. Therefore, the approach used in this study would be useful for the construction of β-oxidation mutant in various strains.

In the beginning of this study, we tried to make a uracil auxotrophic mutant by a traditional method of homologous recombination using a single disruption cassette consisting of the *NAT1* gene flanked by 1 kb homologous regions. Although many transformants (more than 160 colonies) were obtained by NTC selection, it was extremely hard to find a correct integrant of the *NAT1* gene into the *CaURA3* locus with only 13 correct transformants from 120 tested colonies. The finding implies that homologous recombination was overwhelmed by non-homologous recombination in *C. aaseri* SH14. Therefore, targeted integration via homologous recombination in *C. aaseri* SH14 was a problem that needed to be solved. Since CRISPR-Cas9-induced DSB has accelerated homologous recombination in many kinds of yeast [29,38,40], we demonstrated gene knock-in by homologous recombination at specific site of *AOX2* locus by CRISPR-Cas9. The *CaLIP2* cassette without selection marker surrounded with 380 bp of *CaAOX2* 5′ and 3′ flanking regions was co-transformed with the Cas9 vector containing the sgRNA expression cassette targeted to *CaAOX2.* Although a number of ectopic integrations of *CaLIP2* cassette by non-homologous recombination (40%) occurred, the correct integration by homologous recombination (60%) surpassed non-homologous recombination. This efficiency could guarantee genetic manipulation of *C. aaseri* SH14 by homologous recombination.

In mammalian CRISPR-Cas9 systems, off-target effects that related to the lifetime of Cas9 in a cell has been a major problem awaiting a solution [44,45] Although we did not confirm off-target editing in *C. aaseri* SH14, this side effect of CRISPR-Cas9 systems could be minimised in our system because of the instability of ARS, facilitating rapid curing of the Cas9 vector.

In conclusion, we have developed a single plasmid based CRISPR-Cas9 system for efficient genetic manipulation of *C. aaseri* SH14. The system could accelerate the development of various engineered strains capable of producing valuable bio-based chemicals from renewable oils.

## Supplementary Materials

Supplementary materials can be found at https://www.mdpi.com/2076-2607/8/4/526/s1. Table S1: Oligonucleotide used for sequencing and amplification of sgRNAs, Table S2: Plasmid used in this study. Figure S1: Lipase activity of 16 yeasts isolated from compost of empty fruit bunches of palm oil, Figure S2: Utilization of long-chain fatty acids and alkanes by different yeasts and resistance to organic acids, Figure S3: Construction of sgRNA expression vector, Figure S4: Antibiotic sensitivity test of *C. aaseri* SH14, Figure S5: Sequences of ARS1 and ARS2, Figure S6: Sequence of *C. aaseri* codon optimised Cas9 gene, Figure S7: Integration of CaLIP2 overexpression cassette at sgAOX2 via NHEJ, and Figure S8: Effect of Cas9 expression on the growth of *C. aaseri* SH14.
