# Supplementary material for: Genetic Manipulation of a Lipolytic Yeast Candida aaseri SH14 Using CRISPR-Cas9 System
Source: Microorganisms. 2020 Apr 7;8(4):526. doi: 10.3390/microorganisms8040526 (PMC7232369; doi:10.3390/microorganisms8040526)
Supplement: Supplementary file 1 [file microorganisms-08-00526-s001.pdf]

Table S1. Oligonucleotide used for sequencing and amplification of sgRNAs.

Primer name	Sequences 5′ – 3′
TEF-F	GGTCTAGAACCTGGACAAATCGTTAAAC
CYC-R	GCGGCCGCCTTCGAGCGTCCCAAAACCT
sgURA3-1	GGTGGGCTTCAGCTCTCGTCGTTTTAGAGCTAGAA
sgURA3-2	GTCCGTGAGGACGAAACGAGTAAGCTCGTCGGTGGGCTTCAGCTC
sgURA3-3	TTCGTCCTCACGGACTCATCAGGGTGGGTATGAATTATATTAA
sgAOXS-1	GATCAATGTAGACAAGCTTGGTTTTAGAGCTAGAA
sgAOXS-2	GTCCGTGAGGACGAAACGAGTAAGCTCGTCGATCAATGTAGACAA
sgAOXS-3	TTCGTCCTCACGGACTCATCAGGATCAATATGAATTATATTAA
sgAOX2-1	CTGCATTTCCGGCATTCCCAGTTTTAGAGCTAGAA
sgAOX2-2	GTCCGTGAGGACGAAACGAGTAAGCTCGTCCTGCATTTCCGGCAT
sgAOX2-3	TTCGTCCTCACGGACTCATCAGCTGCATTATGAATTATATTAA

Table S2. Plasmid used in this study.

Name	Backbone	Marker	Promoter	Gene	Terminator	origin
P414 *CAS9(TEF1)*	P414	*TRP1*	*ScTEF1p*	*CAS9*	*ScCYC1t*	Reference 28
pNTA	pNTA	*NTC*	*CaGAPp*	*NTA*	*SaGAPt*	This study
pAN-ARS	pAN	*NTC*	*CaURA3p*	*NTA*	*CaURA3t*	This study
pAN-CAS9	pAN	*NTC*	*CaGAPp*	*CAS9*	*CaGAPt*	This study
pAN-CAS9gURA3	pAN-CAS9	*NTC*	*CaTEF1p*	*sgURA3*	*ScCYC1t*	This study
pAN-CAS9gAOXS	pAN-CAS9	*NTC*	*CaTEF1p*	*sgAOXS*	*ScCYC1t*	This study
pAN-gURA3	pAN	*NTC*	*CaTEF1p*	*sgURA3*	*ScCYC1t*	This study
pAN-gAOXS	pAN	*NTC*	*CaTEF1p*	*sgAOXS*	*ScCYC1t*	This study
pAN-gAOX2	pAN	*NTC*	*CaTEF1p*	*sgAOX2*	*ScCYC1t*	This study

Ca : *Candida aaseri, Sc* : *Saccharomyces cerevisiae,* sg: single guide

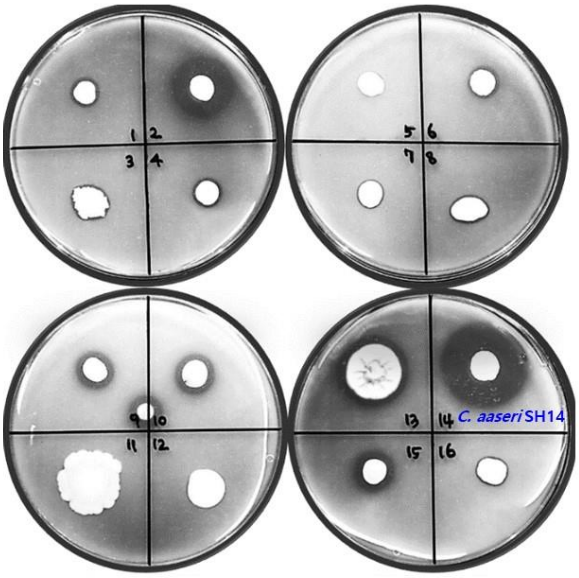

Strain number	Yeast species	Accession No.	Similarity (%)	Lipase activity
1	*Candida sp.*	JQ247717	94.0	+
2	*Cryptococcus laurentii*	FN428921	97.1	+++
3	*Yamadazyma mexicana*	AB365477	97.0	+
4	*Rhodotorula mucilaginosa*	HE660061	95.0	+
5	*Pichia caribbica*	KC111450	94.4	-
6	*Candida sp.*	JQ247716	91.0	+
7	*Pichia caribbica*	KC111450	95.3	+
8	*Pichia caribbica*	KC111450	96.0	+
9	*Rhodotorula mucilaginosa*	KC205168	96.2	++
10	*Sphingobium yanoikuyae*	JN700070	99.0	++
11	*Pantoea agglomerans*	FJ593002	99.2	-
12	*Ochrobactrum intermedium*	AM490610	98.5	-
13	*Uncultured Verticillium*	GU055566	96.2	++
**14**	***Candida aaseri***	**JQ247716**	**94.1**	+++++
15	*Rhodotorula sp.*	JX310560	96.7	++
16	*Candida sp.*	JQ247716	93.4	+

Figure S1. Lipase activity of 16 yeasts isolated from compost of empty fruit bunches of palm oil. Cells were spotted on YNB plate containing 2% glucose and 1% of tributyrin and grown for 5 days.

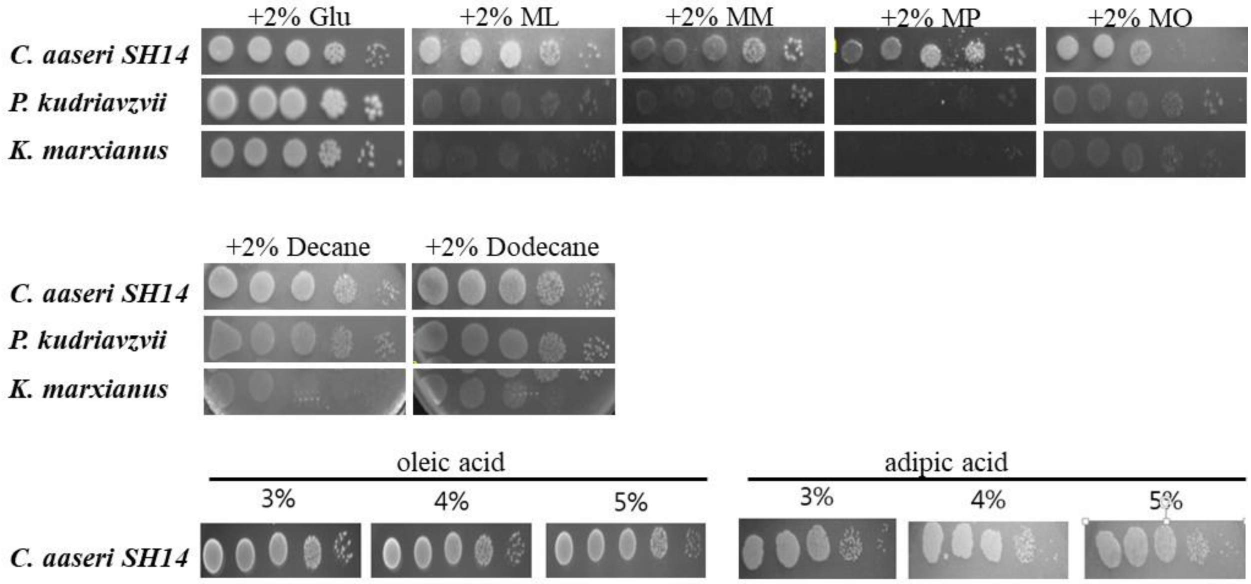

Figure S2. Utilization of long-chain fatty acids and alkanes by different yeasts and resistance to organic acids. Serially diluted yeasts were spotted on YNB plate containing 2% of the indicated carbon sources and grown for 5 days. ML; methyl lauric acid, MM; methyl myristic acid, MP; methyl palmitic acid, MO; methyl oleic acid.

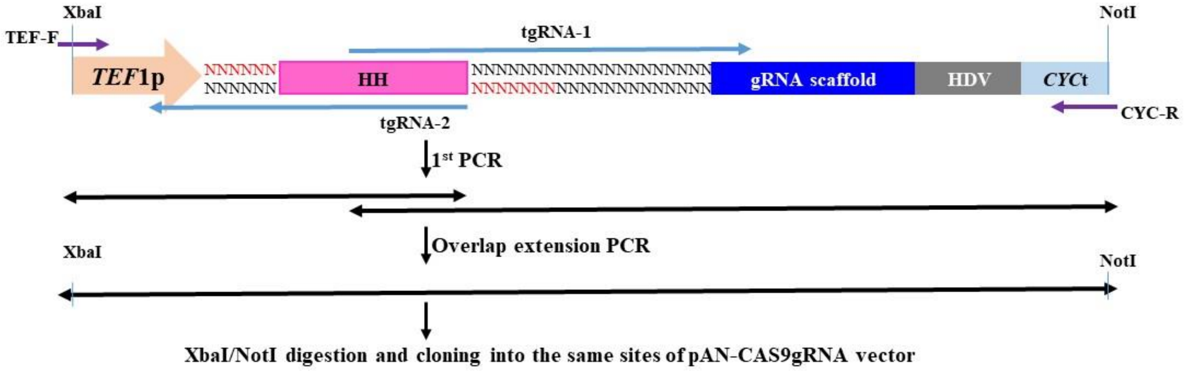

Figure S3. Construction of sgRNA expression vector. NNNNNN indicate 6 bp complementary sequence required for release of HH from sgRNA. Two fragment were amplified using TEF-F/tgRNA2 and tgRNA1/CYC-R primers, respectively and annealed into a single fragment by overlap extension PCR using TEF-F and CYC-R primers.

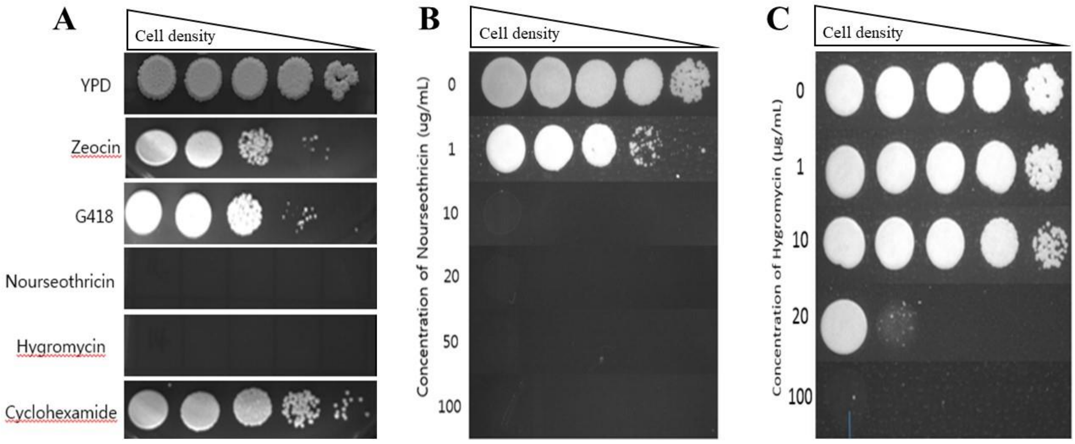

Figure S4. Antibiotic sensitivity test of *C. aaseri* SH14. Serially diluted *C. aaseri* SH14 was spotted on YPD plate containing 100 µg/mL of antibiotics and grown for 5 days **(A)**. Determination of minimum inhibitory concentration for NTC **(B)** and hygromycin **(C)**.

>ARS1 (1265 bp)
CTGCAGCCCGGGGGATCAATCCAACTTAAAGTTTCTCTTTGTTGATATGGGTGGTAAAGTTAGT
GAATTTAAAGAAGAGTTCAAGAAGTTAGATTTGTACGCCCCTAATGGTGTGGATATTGTCATTC
ATAATGCTGGTATCAGTGGTCCTAACTTCTTTGTTAAATCTACTGAATACGATGTAGAATCTGCA
CTTCCAGTAGTTGCAATCAACTATCTTGGTACTGTGAAATTGTATCAAGCTGTTTACCCATACCT
TTTCAAAGGAAATGGAACAAAAAAGCTCATTTTAACAAGCTCACTTGCTAGTTCTATGGGACA
AATGCCTTTTGGAAGTAATACTTATGGAGCTTCTAAAGCTGCTGTCAACCATTTTGGTGTCCAA
ATTGCTACTGAACATCAAAATTCAGACAATCCTCTTATTAAAAATTCTATCACTGTATTATTACA
TCCCGGTCTTGTTCTTACTGATATGGCTGCCATGGAAGGAGACTCTGGCTCTTCGGATACTACA
CCTTCTTGTGAATTAATTACACCAGATGTATCTGCAAAAGGCACTTTAGATTTGGTTGCACGTCT
TACCTCAAAAGACAATGGTAAGTTTTTCGATTACCAAGGAAACAACATAAGTTTGTAAGTCCA
CTAGTGGAGGTCATATTCCATTTTTGTTTAAGGTATTTCACTTGTTGAAATCATTTGTATGATAAA
TGAAACATCAAGAGTCGTGTATGGTCATTTGAATAATATCTAGTAATAATCCACTAAAAGACCA
ATTTTCCTTCTTCATAGTTTTCTAATAGATTCCAATTACTTTATTTATAGCATGAGTTTTTTTTTCTG
TATTTTTAAAGCTCTTTGAAATGAATTGTTAAAACTTCTTGATATCTTTACTCTTTATTTTCTCTGT
TGTGAAAATCACAATTTAAAAAATTTTCTACTCTCATACTGTTACTGAATTCTTCACTCATCAAT
GAAAATTCCATTTCTCTATAAAATAATCAGACCTCTCACACCATATCAATAAATAGACGTACCT
TCACTAGGTGAATATAGAAGCAGTATATACAGTATGGCATCCTCTGGAGAACCTATTCGGATGG
GACTTACTACCCTATATTATGGTTCCTCGCGAAAAATCTGGGGAAATTATCACACCATGTTCAG
TACAAGAACCATTGCCTCGAGATTGTTTGGAACTAGATTCGTTTCCCATTCTCTTTCAGTGGTCA
AATATGATAAGAGTTCCACCTCATTGAAGATCCACT

>ARS2 (789 bp)
AGATATCATGTGGAAGGAAAACATGATGTTGTTTATATTTCAGAACCCTTTGAAATCAAAGTTA
GAGAGTTGGATGTCCCTACATCATCAGATGAAGTTACACTGTTTTGCAAATCAATTGAAATACT
TAACTGATAAAGTATTGGAATTAGATCTGATTTATCACAAAATCGAATCAATGAACTCTAACAT
TTTAGTTATTTTTGAAAGATTAACTAAATTGATTTCATTATCAACCAATATTAAGATTAATGAAA
AGGTATTTTTGAATGAAGATATGACTTTGATTGACTTGTCAGCCAAATTAATTCATATGAAAAA
AGTATTGGAAGATTTATCATTCCAAAAATACAATTTAGAAAAAAAAATGGAATAAAATCCTGT
AGATAGAATACATTATACAAATAACTAGAAGAAATATAAAAAGTATAACAAAACATGTTGAAA
TAATAATTTGTAGAAATTATCTCTTTGAAAAAGTAAGATTTGTAAAAATCACTAAAAGGGTAAG
TAAACCACGAAGCAAAAAATATAGAATATAATCACTTCAAAGAGTCACGGAAACATATCTGTC
CTCGCgTGCCCCAAACATTATTCATTGACTAAACATTCTCTAATAAATAACTATTTACATTACTCC
TTATCTTCTTTGCCATTTCACTGCAATGTTCAATAGTGTTATCCAAATCCTTTAAGTCACCATTAA
CCCAGATACCAACAATTTCATTCTTCAAGCACATGAAACTAACTAAACAATCGATGTCTAATTG
CAGATCCCCCGGGCTGCAG

Figure. S5. Sequences of ARS1 and ARS2. Yellow boxes indicate the core regions of *ARS1* and *ARS2* for the replication and stability of plasmid.

ATGGATAAGAAATACTCAATAGGCTTAGATATTGGCACAAATAGCGTCGGATGGGCGGTGATC
ACTGATGAATATAAGGTTCCGTCTAAGAAGTTCAAGGTTTTGGGAAACACAGACCGCCACAGT
ATCAAGAAGAATCTTATAGGGGCTCTTTTATTTGACAGTGGAGAGACAGCGGAAGCGACTCGT
CTCAAACGGACAGCTCGTAGAAGGTATACACGTCGGAAGAATCGTATTTGTTATCTACAGGAA
ATCTTCTCAAATGAGATGGCGAAAGTAGATGATAGTTTCTTTCATCGACTTGAAGAGTCTTTCT
TGGTGGAAGAAGACAAGAAGCATGAACGTCATCCTATCTTTGGAAATATAGTAGATGAAGTTG
CTTATCATGAGAAATATCCAACTATCTATCATTTGCGAAAGAAATTGGTAGATTCTACTGATAAA
GCGGATTTGCGCTTAATCTATTTGGCCTTAGCGCATATGATTAAGTTTCGTGGTCATTTCTTGATT
GAGGGTGATCTAAATCCTGATAATAGTGATGTGGACAAACTATTCATCCAGTTGGTACAAACCT
ACAATCAATTATTTGAAGAAAACCCTATTAACGCAAGTGGAGTAGATGCTAAAGCGATTCTTTC
TGCACGATTGAGTAAATCAAGACGATTAGAAAATCTCATTGCTCAGCTCCCCGGTGAGAAGAA
AAATGGCTTATTTGGGAATCTCATTGCTTTGTCATTGGGTTTGACCCCTAATTTCAAATCAAATT
TTGATTTGGCAGAAGATGCTAAATTACAGCTTTCAAAAGATACTTACGATGATGATTTAGATAA
CTTATTGGCGCAAATTGGAGATCAATATGCTGATTTGTTCTTGGCAGCTAAGAATTTATCAGATG
CTATCTTACTTTCAGATATTCTAAGAGTAAATACTGAAATAACTAAGGCTCCCCTATCAGCTTCA
ATGATTAAACGCTACGATGAACATCATCAAGACTTGACTCTTTTAAAAGCGTTAGTTCGACAA
CAACTTCCAGAAAAGTATAAAGAAATCTTTTTTGATCAATCAAAGAACGGATATGCAGGTTAT
ATTGATGGGGGAGCTAGCCAAGAAGAGTTCTATAAGTTCATCAAACCAATCTTAGAAAAGATG
GATGGTACTGAGGAATTATTGGTGAAACTAAATCGTGAAGATTTGTTGCGCAAGCAACGGACC
TTTGACAACGGCTCTATTCCCCATCAAATTCACTTGGGTGAGTTGCATGCTATTTTGAGAAGAC
AAGAAGACTTCTATCCATTCTTAAAAGACAATCGTGAGAAGATTGAAAAGATCTTGACTTTTC
GAATCCCTTATTATGTTGGTCCATTGGCGCGTGGCAACAGTCGTTTTGCATGGATGACTCGGAA
GTCTGAAGAAACAATTACCCCTTGGAATTTTGAAGAAGTTGTCGATAAAGGTGCTTCAGCTCA
ATCATTTATTGAACGCATGACAAACTTTGATAAGAATCTTCCAAATGAGAAAGTTCTACCAAA
ACATAGTTTGCTTTATGAGTATTTTACGGTTTATAACGAATTGACAAAGGTCAAATATGTTACTG
AAGGAATGCGAAAACCAGCATTTCTTTCAGGTGAACAGAAGAAAGCCATTGTTGATTTACTCT
TCAAAACAAATCGAAAAGTAACCGTTAAGCAATTAAAAGAAGATTATTTCAAGAAAATAGAA
TGTTTTGATAGTGTTGAGATCTCAGGAGTTGAAGATAGATTTAATGCTTCATTAGGTACTTACCA
TGATTTGCTAAAGATCATTAAAGATAAAGATTTCTTGGATAATGAAGAGAATGAAGACATCTTA
GAGGATATTGTCCTTACATTGACCTTATTTGAAGATAGGGAGATGATTGAGGAAAGACTTAAA
ACATATGCTCACCTCTTTGATGATAAGGTGATGAAACAGCTTAAACGTCGCCGTTATACTGGTT
GGGGACGTTTGTCTCGAAAATTGATTAATGGTATTAGGGATAAGCAATCTGGCAAAACAATATT
AGATTTCTTGAAATCAGATGGTTTTGCCAATCGCAATTTCATGCAGTTGATCCATGATGATAGTT
TGACATTCAAAGAAGACATTCAAAAAGCACAAGTGTCTGGACAAGGCGATAGTTTACATGAA
CATATTGCAAACTTAGCTGGTAGCCCTGCTATCAAGAAAGGTATTTTACAGACTGTAAAAGTTG
TTGATGAATTGGTCAAAGTAATGGGGCGGCATAAGCCAGAGAACATCGTTATTGAAATGGCAC
GTGAAAATCAGACAACTCAAAAGGGCCAGAAGAACTCGCGAGAGCGTATGAAACGAATCGA
AGAAGGTATCAAAGAGTTAGGAAGTCAGATTCTTAAAGAGCATCCTGTTGAAAATACTCAATT
GCAAAATGAAAAGCTTTATCTCTATTATCTCCAAAATGGAAGAGACATGTATGTGGACCAAGA
ATTAGATATTAATCGTTTAAGTGATTATGATGTCGATCACATTGTTCCACAAAGTTTCCTTAAAG
ACGATTCAATAGACAATAAGGTCTTAACGCGTTCTGATAAAAATCGTGGTAAATCGGATAACG
TTCCAAGTGAAGAAGTAGTCAAAAAGATGAAGAACTATTGGAGACAACTTCTAAACGCCAAG
TTAATCACTCAACGTAAGTTTGATAACTTGACGAAAGCTGAACGTGGAGGTTTGAGTGAACTT
GATAAAGCTGGTTTCATCAAACGCCAATTGGTTGAAACTCGCCAAATCACTAAGCATGTGGCA

CAAATTTTGGATAGTCGCATGAATACTAAGTACGATGAAAATGATAAACTTATTCGAGAGGTTA
AAGTGATTACCTTAAAGTCTAAATTAGTTTCTGACTTCCGAAAAGATTTCCAATTCTATAAAGT
ACGTGAGATTAACAATTACCATCATGCCCATGATGCGTATCTAAATGCCGTCGTTGGAACTGCT
TTGATTAAGAAATATCCAAAACTTGAATCGGAGTTTGTCTATGGTGATTATAAAGTTTATGATGT
TCGTAAGATGATTGCTAAGTCTGAGCAAGAAATAGGCAAAGCAACCGCAAAGTATTTCTTTTA
CTCTAATATCATGAACTTCTTCAAGACAGAAATTACACTTGCAAATGGAGAGATTCGCAAACG
CCCTCTAATCGAAACTAATGGGGAAACTGGAGAAATTGTCTGGGATAAAGGGCGAGATTTTGC
CACAGTGCGCAAAGTATTGTCCATGCCCCAAGTCAATATTGTCAAGAAGACAGAAGTACAGA
CAGGCGGATTCTCCAAGGAGTCAATCTTACCAAAAAGAAATTCGGACAAGTTGATTGCTCGTA
AGAAAGACTGGGACCCAAAGAAGTATGGTGGTTTTGATAGTCCAACGGTAGCTTATTCAGTCC
TAGTGGTTGCTAAGGTGGAGAAAGGGAAATCGAAGAAGTTAAAATCCGTTAAAGAGTTACTA
GGGATCACAATTATGGAAAGAAGTTCCTTCGAGAAGAATCCGATTGACTTTTTAGAAGCTAAA
GGATATAAGGAAGTCAAGAAAGACTTAATCATTAAACTACCTAAATATAGTCTTTTTGAGTTAG
AAAACGGTCGTAAACGGATGTTGGCTAGTGCCGGAGAATTACAGAAAGGAAATGAGTTGGCT
TTGCCAAGCAAATATGTGAATTTCTTATATTTAGCTAGTCATTATGAAAAGTTGAAGGGTAGTCC
AGAAGATAACGAACAAAAACAATTGTTTGTGGAGCAGCATAAGCATTATTTAGATGAGATTAT
TGAGCAAATCAGTGAGTTCTCTAAGCGTGTTATTTTAGCAGATGCCAATTTAGATAAAGTTCTT
AGTGCATATAACAAACATAGAGACAAACCAATACGTGAACAAGCAGAAAATATCATTCATTTA
TTTACGTTGACGAATCTTGGAGCACCCGCTGCTTTCAAATATTTTGATACAACAATTGATCGTA
AACGATATACGTCTACAAAAGAAGTTTTAGATGCCACTCTTATCCATCAATCCATCACTGGTCT
TTATGAAACACGCATTGATTTGAGTCAGCTAGGAGGTGAC

Figure S6. Sequence of *C. aaseri* codon optimised Cas9 gene.

AGTATCAAATTTCATTAATGAATAAGTTTCAACTTGTAAATCACATAAAATCTCTACCGCTTTAT
CCAAATCATTTTTCTTTGTTAAATAAATATCACTCAAATTACGTGATACAATTGCCCTTTCTACTT
CAATAAAGATTTTTTTATCACTAACAGTTCGAATAGTTTCAATCAATTTAATCTTTAATTCTAATT
CTTTATGATTATTCTCATCTAAACTGGCCATTTTCTCAATTATATGTGCAATAAACACTTGGATAC
TGCTTTTCATTTGTCCATGCTTCTTACATAAGATGGGAATCAATTCATTCATGTATTCCCAGTCTG
ATTCTGCCAATTTGTCCACAATGGCTATCAATACTCTTTTACTACTTACAAGATCTGACAGTTGC
CTTACTTGTTTTTCTAAAGAAAGAAGACGGTCTACCGCTTCCTTGTATGGAAGCAGGGTGATTT
CCGGGAGTTGTTTGTCCAAGATGGACGTGTAGTCCTTCTCTGCTTTTAAAGGATCTTCTCTAGA
CATGTTGAGTGGCGAAACTTTGTGGTGATTTCTTGAAAAGCGCGATATTTGAAGCCAAATTTCA
ACACAAGAAACATGAGTAGGACACGCCTTTTTTATTATTTAAAGATTCTAAATAGTTAATAAAG
GCAAATACAATTGATATTAGTTATGGTGGAAATTGGTACCGATGGGAAGTTGAAAGTAATTACA
AAAAGAGTACCCGCGGATTTGATTTGGGCCAATCATGGCCAAGTACCCCTGTCAAATTATCATT
AATTCACATTCATTTTGTGGGGAAAAAATTGTATGTGGGGACATTATTCAAGTATAAATACCAA
TTGAAATAGCAGGAAATCATTTTTCAATATGTTTGTCACTAATAAAATTGATAGTAGTGAAGCA
CCACGTCCTAGCGAGTTGATTTCCAACGAAAGAGCTGCATTTCCGGCATTCGGCGAAACTTTG
TGGTGATTTCTTGAAAAGCGCGATATTTGAAGCCAAATTTCAACACAAGAAACATGAGTAGGA
CACGCCTTTTTTATTATTTAAAGATTCTAAATAGTTAATAAAGGCAAATACAATTGATATTAGTTA
TGGTGGAAATTGGTACCGATGGGAAGTTGAAAGTAATTACAAAAAGAGTACCCGCGGATTTG
ATTTGGGCCAATCATGGCCAAGTACCCCTGTCAAATTATCATTAATTCACAT*ACCTGGACAAATC*
GTTAAACGGCTACCTTTTAAATATATAGAAATATAAACCCCACTATGGAATTTGAATAACTAATAACC
CTGAGCCATGAATAGACTGATAATCAATTGGTTATCTCTTTCTTACCAAGCTAGAACTTCACTATACT
ATGACTTTCACGTAGAAATGTCCCCAAAAAAAAAGTCGATCTCATTAAATTCAGCAACACCCTCTTT
TAAAATACAAACCACTAGGGTGTGCAATTCCATGTAACAAATTGAAATTTAAATTGAAATTTGCAAC
CAAGCCTTCAAAATTAACGAATTTTCATGTTTTTCTATTAACTAAAACATTTAATTAAGCTTCAAATA
AAACTTGAACACTTTGGTCTAAACCTTGTCTATTTATTCTATATAATTCTATTATTAAAATTTCCATAC
ACGCACACCTTATCATTGCAGTGAAAAATTTTTCAAGGACGCTCATCTTAAATATCTGAAAAATATA
AATTACAGCAATCCGCCCCTTTGAAAATTTTTTTCCCTCTCCATACTTGTTCTTTTTTGTTTTAAAGTC
*AATTAATATAATTCATAGGATCC*ATGATCTGTTCATTCTTTAGACTATTGACCATTGTAACTTTGGT
TATTGCTGCTCCTACCACTTTAGTTCCTCCAACTGAAGATCCTTTCTATACTGCACCAAAGGGCT
TCGAATCAGCAGAGTTAGGTACTGTTTTGGCTTATAGAAACACTCCAGCTCCAATCAGAAGTAT
TTATTTTGAAGTTAATATCAAAAACTCATGGCAATTGTTAGTCAGGTCTTCTGATTCATTTGGTA
ATCCTTCAGTAGTTGTAACTACTGTTTTTGAACCATTTAATGCTGATCCTTCCAAGTTAGTTTCTT
ATCAAGTTGCTCAAGATTCTGCATATCTTGATTGTTCACCATCATATTCCTTCATGAATGGAGGT
GGTCTTTCTACTATTAACAATCAAATTGAGACTGTTTTAATTCAAACAGCATTAGACCAAGGTT
ATTATGTTGTTTCTCCAGATTATGAAGGATTGAAATCGGCTTTCACCGGTGGTATTCAAGCTGGT
CATGGTACATTGGATTCCATTAGAGGTGCTTTATCCAGTAGTAACATCACTGGTGTTAAAAAGG
ACGCAGATACTATTCTTTGGGGTTATTCTGGAGGTTCTTTAGCTAGTGGATGGGCTGCAGCTTTA
CAACCAACTTATGCACCAGAATTGGCTTCCAACTTACTTGGTGTTGCTTTAGGTGGATGGGTTA
CCAATATTACTGCTACTATAACAAGTGTTAGTGGTACCATATTCTCTGGATTGGGTGCTATGGGA
ATGGCTGGTTTAAGTAATGAGTACACCGATTTATACGGTTACCTTAAGACTGCTATGCCAGCAG
ATAAATATGAAGAATTCACTAAAGCTTATTCAATATGTGCTGCTGAAGCTCTTATTGAATATAAT
TTTGATGATTGGTTTGAAGGCGAAGATAGATACTTTACCGATGGCTTTAAAGTTTTGAATGAAG
AACCGACATATTCCATCATTCGTAACAATACATTGGGTTTAATTGCTGGTCAGATGCCAGAAAT
TCCTGTTTTCGTTTACCATGGAACTCTCGACCAGATCGTACCATATGATCAAGCTGAAAGGGTT

TATGATATTTGGTGTGATGCTGGTATTAAATCTTTTGAATTTGCTACTGATTTAACTGCTGGTCAT
ATCACTGAGCTTGTACAAGGTAGTGGTGCAGCCTTTGGATGGATCAAAGGCATGTTTGAAGGA
ACTAAAAAACCAGTTTCTGGTTGTAGAAAAACACCTAGAATTTCAAACTTACTTTACCCAGGT
ACTGTAAGATCTGTTACTGACGTTGTTGGTGCTTTGCTTGATAATATCTTAGGATTTGATATTGGT
CCAAATGGTGAAAACCTTATTGTTGAAAACAACAGCGTCATCAGTAAAGCTAATTCTACTAGA
AGTGAACATCATCACCATCACCACTAGGTCGAC**CTAGCCGTGGAACATAAATTATAAGTTTT
ATAGTATACTTAGTGTTTTTATATGAATTTATAGTTTGTGGATTTTAAAAATTTCATGTCAAT
GGCTTCAATTGACTGTGTAATTGTAGTATATAATTGTAGTTGGTTTGATCAATGATTGAAAG
GCGCGATTAGATTAATGTTTTTATTACATAAACGATATTACGATAT**TCTTGAAGGCAGCCAGA
AAAACTCCGAAATTATTTTAAAGATTTCCAGCCAATTGGAAAAAGATCCGATTCTTCAGAGCT
CTTTCGGAGAATATGATATGCTGACAACACAACAGCGGGAATTGACGGCTTTGAGAATAGATA
GACTCACTAATTACCGAGAATTAGAATCTATAGATGATTTTTTCACTCGATTAAATCTTATCACC
ATTTATGATCCCAGTTTAGGAATTCGAATCTCAATCAATTTAGGCTTGTTTTTAAACTGTATTAA
AGGTAATGGAACTGCTAGTCAGGTGGAATATTGGTGTAATCGCAAGGAGGCACTGATTTTAAA
GCAAATTTATGGATGTTTTGCCATGACAGAACTAGGACATGGTTCTAATGTACCACGGACTAAA
ATTCATCAGTTTCTTGAAGGCAGCCAGAAAAACTCCGAAATTATTTTAAAGATTTCCAGCCAAT
TGGAAAAAGATCCGATTCTTCAGAGCTCTTTCGGAGAATATGATATGCTGACAACACAACAGC
GGGAATTGACGGCTTTGAGAATAGATAGACTCACTAATTACCGAGAATTAGAATCTATAGATG
ATTTTTTCACTCGATTAAATCTTATCACCATTTATGATCCCAGTTTAGGAATTCGAATCTCAATCA
ATTTAGGCTTGTTTTTAAACTGTATTAAAGGTAATGGAACTGCTAGTCAGGTGGAATATTGGTGT
AATCGCAAGGAGGCACTGATTTTAAAGCAAATTTATGGATGTTTTGCCATGACAGAACTAGGA
CATGGTTCTAATGTA

Figure S7. Integration of CaLIP2 overexpression cassette at sgAOX2 via NHEJ. The blue box indicates the 5′ repetitive sequence and the yellow box indicates the 3′ repetitive sequence. The red box is a PAM sequence and gray box is a recognition site for DNA cleavage by CRISPR-Cas9. Underlined sequences indicate *Ca*LIP2 gene sequence. *TEF1* promoter is an italic character before *CaLIP2* sequence and *CYC1* terminator sequences located downstream of *CaLIP2* with bold character.

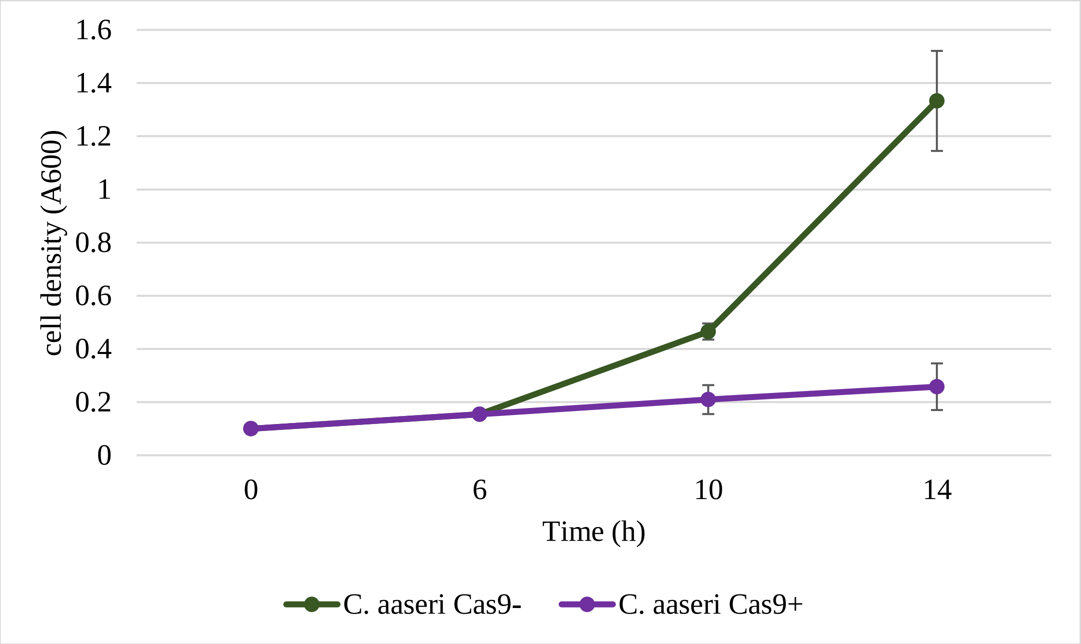

Figure S8. Effect of Cas9 expression on the growth of *C. aaseri* SH14. *C. aaseri* SH14 harboring a plasmid free of Cas9 (Cas9-) and a plasmid with Cas9 (Cas9+).
